# Homology Modeling of Sesame Allergenic Protein and Prediction of B-Cell Linear Antigenic Epitopes Using Immunoinformatic Tools

**DOI:** 10.3390/foods14234158

**Published:** 2025-12-03

**Authors:** Xiuli Ma, Fang Wang, Ning Yu, Jiukai Zhang, Xiaoxuan Wang, Meng Xie, Yiqiang Ge, Ying Chen

**Affiliations:** 1 School of Food Engineering, Yantai Key Laboratory of Nanoscience and Technology for Prepared Food, Yantai Engineering Research Center of Food Green Processing and Quality Control, Ludong University, Yantai 264025, China; 2 Chinese Academy of Inspection and Quarantine, Beijing 100176, China; yu962925@126.com (N.Y.); zhjk_caiq@163.com (J.Z.); 3 China Rural Technology Development Center, Beijing 100045, China; 68511009@163.com

**Keywords:** sesame allergen, bioinformatics, B-cell antigenic epitope, structure

## Abstract

Sesame (*Sesamum indicum* L.) has emerged as a significant food allergen and is now classified among the “Big Eight” allergens due to its increasing prevalence and potential to cause severe allergic reactions, including anaphylaxis. Seven sesame allergens have been identified; however, their structures and epitopes have not been thoroughly studied. In the present study, we predicted the tertiary structures of these seven sesame allergens and identified the B-cell epitopes using immunoinformatic tools, suggesting them as potential targets for allergen immunotherapy. Consequently, homology modeling and tertiary structure prediction were performed for the seven allergens with unknown structures. A total of 62 peptides were identified through prediction analysis. Twenty-eight out of the 62 predicted epitopes are located in regions that are positionally conserved with previously reported epitopes in homologous allergens. They share certain key amino acids. The spatial distribution of some predicted B-cell linear epitopes is depicted, providing multiple perspectives. The predicted consensus epitopes and structures can serve as suitable candidates for designing immunotherapeutic vaccines.

## 1. Introduction

Over recent decades, global food allergy prevalence has risen markedly with no signs of abatement [1,2]. Exposure to minute quantities of common foods can trigger rapid allergic reactions ranging from mild symptoms to life-threatening anaphylaxis, elevating this condition to a paramount public health concern that demands improved prevention, diagnosis, and treatment strategies [2,3]. Sesame (*Sesamum indicum* L.) is a valuable oilseed crop with numerous nutritional benefits, containing a diverse range of bioactive compounds. However, sesame is also recognized as a potent allergen capable of inducing severe reactions in sensitized individuals [4]. Reflecting its epidemiological significance and risk severity, regulatory bodies including Health Canada, the European Union, and the Joint Food and Agriculture Organization (FAO)/World Health Organization (WHO) Expert Committee have designated sesame a priority allergen [5]. Notably, the FAO/WHO Expert Consultation on Risk Assessment of Food Allergens formally incorporated sesame into the expanded “Big Nine” allergens (replacing soybean), mandating its inclusion in global allergen labeling regulations [1].

Antigenic epitopes represent the immunological basis for eliciting food allergy responses triggered by allergenic molecules. They are integral components that play a crucial role in facilitating antibody binding processes. Therefore, the identification of epitopes is a necessary prerequisite for understanding the sensitization mechanisms of allergens, including responses to various processing methods, such as roasting, and potential cross-reactivity [6,7,8]. Research on the localization of allergenic epitopes is vital for comprehending allergens, as the prediction and mapping of these epitopes provide essential insights into allergenic antigenic sites [7,8,9,10].

Immunodominant B-cell epitopes are primarily responsible for the incitation of the early-phase allergic reactions [11]. These immediate, IgE-mediated responses are often the most acute and dangerous clinical manifestations of food allergies. Therefore, identifying and characterizing these specific linear epitopes is a critical and foundational step toward the rational design of hypoallergens or multi-epitope allergy vaccines aimed at blocking or reducing IgE binding. While T-cell epitopes are crucial for the late-phase reactions and for modulating long-term immune tolerance [12], and conformational epitopes certainly contribute significantly to allergenicity, our study’s primary objective was to identify the fundamental sequential components that trigger the initial antibody response. This computational approach serves as a pragmatic and established starting point. The linear epitopes identified here can serve as a basis for future studies, including the experimental validation and the structural analysis of more complex conformational epitopes. Thus, identifying epitopes of food allergens enhances our understanding of allergy mechanisms and facilitates the development of hypoallergenic foods and the design of immunotherapies.

The primary allergenic proteins in sesame include two 2S albumins (Ses i 1 and Ses i 2), a 7S vicilin (Ses i 3), two oleosins (Ses i 4 and Ses i 5), and two 11S globulins (Ses i 6 and Ses i 7) [13,14,15,16,17,18]. However, despite their significance, the specific epitopes and detailed structures of most sesame allergens remain largely uncharacterized [18]. Currently, very few studies have reported potential IgE-binding epitopes and structures for these sesame allergenic proteins. To date, only two studies have identified the most likely allergenic epitopes within Ses i 2 and Ses i 4 using a method based on overlapping peptide libraries, reporting peptide sequences such as SRQCQMRHCM, QCQMRHCMQW, NQGQFEHFREC, PSTTQI, TSGAFGLTG, and KRGVOEGTLYNR [19,20]. This significant gap in knowledge highlights a critical area for future research, especially considering our previous study investigated the responses of the structure and IgE binding of sesame allergens to different roasting treatments [21]. Understanding how processing affects epitopes is essential but currently hindered by the lack of comprehensive epitope and structure data for sesame allergens. The current study constitutes the first comprehensive, purely computational predictive analysis targeting B-cell epitopes across multiple major sesame allergens. Although the Ses i 5 study validated specific epitopes both computationally and experimentally [22], our present work aims to provide a broad, foundational map of potential B-cell epitopes for the entire sesame allergen family, filling a distinct and significant gap in the literature.

Various methods are employed in allergenic epitope research, including the selection of allergenic peptide segments using allergenic serum following enzymatic digestion, the preparation of monoclonal antibodies, the design of overlapping peptides based on allergenic amino acid sequences, two-dimensional immunoblots, and mass spectrometry for the determination of conformational and linear epitopes [22]. The complexity of antigenic determinant clusters within proteins presents significant challenges in localization analysis. These methods are often cumbersome, labor-intensive, and less efficient. Therefore, relying solely on experimental methods for epitope localization poses considerable challenges. The utilization of bioinformatics databases and software for epitope prediction effectively narrows the scope of antigenic epitope screening, thereby reducing the experimental workload and enhancing efficiency [23,24]. The immunoinformatic methods are the advantageous techniques to identify and screen immunodominant epitopes prior to experimental studies in order to save cost and time [25].

Specifically, B-cell linear epitope prediction leverages protein sequence data through algorithms that analyze physicochemical properties (e.g., hydrophilicity, flexibility), structural features (surface accessibility, secondary structure), and antigenicity parameters. Consensus analysis using tools such as BepiPred, DNAStar, and SOPMA further improves prediction accuracy [26]. Crucially, identified B-cell epitopes enable non-covalent antibody binding and facilitate immunotherapy design through epitope-engineering approaches. To date, high-resolution structural information for most sesame allergens remains lacking, which limits in-depth understanding of their molecular characteristics and allergenicity. To fill this knowledge gap, this study employed the state-of-the-art deep learning tool AlphaFold3 to predict the tertiary structures of key sesame allergens, thereby providing a fundamental structural basis for future immunological research [27].

This study used bioinformatic tools to predict B-cell antigenic epitopes of sesame allergens through the use of multi-parameter software. To better understand their potential immunological relevance, the predicted epitopes were visualized on 3D models generated by AlphaFold3. This structural analysis focused on evaluating their surface accessibility and spatial clustering, key determinants for identifying potential immunodominant hotspots and conformational epitopes. This research offers significant references for the accurate detection, diagnosis, and treatment of sesame allergies.

Indeed, bioinformatics tools have proven invaluable in characterizing allergens from various sources. For instance, several studies have successfully applied epitope prediction algorithms and homology modeling to identify potential allergenic regions in major walnut and soybean allergens, providing crucial insights into their allergenic potential [28,29]. In the present work, seven sesame allergens with unknown structure were modeled by homology modeling with AlphaFold3. B-cell epitopes were identified bioinformatically, and the predicted epitopes were displayed in the modeled tertiary structures. Collectively, this work establishes a foundation for advancing precise detection methods, diagnostic tools, and epitope-targeted immunotherapies for sesame allergy management.

## 2. Materials and Methods

### 2.1. Allergen Amino Acid Sequence Retrieval

The full-length translated sequences available in the public databases are considered to represent the mature, functional forms of these proteins. The allergen sequences were retrieved from the Uniprot database (https://www.uniprot.org/), and the detailed information of the seven sesame allergens is shown in Table S3. Prior to analysis, signal peptides, as annotated in UniProt, were removed from the full-length sequences to obtain the mature protein sequences for all allergens. Amino acid sequences of Ses i 1–7: Q9AUD1, Q9XHP1, Q9AUD0, Q9FUJ9, Q9XHP2, Q9XHP0, and Q9AUD2 are shown in Table S2.

### 2.2. Physicochemical Properties Analysis

The average Grand Average of Hydropathicity (GRAVY) values for the seven sesame allergenic proteins were predicted using ProtParam tool (https://web.expasy.org/protparam, accessed on 1 July 2024). To elucidate the biophysical and potential immunological characteristics of the allergens, a series of predictive analyses were performed using the Protean subprogram within the DNASTAR Lasergene software suite v7.1 (DNASTAR Inc., Madison, WI, USA). Fundamental properties including molecular weight (MW), theoretical isoelectric point (pI), and molar extinction coefficient were determined. Subsequently, several structural and antigenic features were predicted: the antigenic index was calculated using the Jameson–Wolf method (combines hydrophilicity, surface accessibility, and flexibility predictions to identify likely antigenic determinants); hydrophobicity profiles were generated via the Hopp-Woods and Kyte-Doolittle methods (identify hydrophobic/hydrophilic regions based on amino acid hydropathy scales); surface accessibility was evaluated with the Emini method (calculates the probability of a region being on the protein surface); and backbone flexibility was assessed using the Karplus-Schulz method (predicts chain mobility based on the normalized B-factors of amino acids in known protein structures) [30,31,32,33,34,35,36].

### 2.3. Prediction of the Secondary Structure of Sesame Allergenic Proteins

The Protean subprogram in DNAStar bioinformatics software was used to predict α-helices, β-sheets, β-turns, and random coils using the Chou-Fasman, Garnier-Robson, and Deleage-Roux methods. The SOPMA bioinformatics tool (http://npsa-pbil.ibcp.fr/cgi-bin/npsa_automat.pl?page=npsa_sopma.html, accessed on 1 November 2024) was also utilized, which incorporates the GOR method, double prediction methods, Levin homology prediction method, PHD method, and CNRS’s SOPMA method to predict secondary structures. The combined results of these five methods were obtained. Ses i 1~7 sequences were submitted to the SOPMA web server, with “Number of conformational states” set to “4 (Helix, Sheet, Turn, Coil),” “Similarity threshold” set to “8,” and “Window width” set to “17” [31,32].

### 2.4. Prediction of Antigenic Epitopes of Sesame Allergenic Proteins

#### 2.4.1. BepiPred Prediction

The BepiPred 1.0 Server (http:/www.cbs.dtu.dk/services, accessed on 1 October 2024) utilizes Hidden Markov Models (HMM) and hydrophilicity parameter scoring to predict linear B-cell binding epitopes. To use the BepiPred 1.0 Server, Ses i 1~Ses i 7 sequences were submitted, and default values were selected. BepiPred software scores peptides according to hydrophilicity values, secondary structures and the probability of an amino acid being located in certain positions as compared to other mapped B cell epitopes. Peptides displaying scores higher than 0.35 may be, therefore, considered putative B cell epitopes [28,36].

#### 2.4.2. Integrated Prediction

The characteristics of proteins (hydrophobicity, flexibility, surface accessibility, etc.) were combined with secondary structure predictions to perform a comprehensive analysis. Various software tools were used to predict hydrophilicity, surface accessibility, flexibility, and antigenic index in different segments of the sequences [36]. Suitable segments with positive hydrophobicity and antigenic indices, surface accessibility greater than 1, and flexibility were selected as potential linear B-cell epitopes of the protein.

The results from the three software tools were combined, and regions with overlapping predictions from at least two of them were considered as epitope candidates. Specifically, we first compiled and aligned all predicted sequences from the three independent tools against the full-length protein to identify regions of overlap. A sequence was then defined as a “consensus region” only if it was part of a continuous sequence identified by at least two of the three tools, thereby systematically discarding any prediction made by only a single tool. To resolve discrepancies in window lengths and merge overlapping predictions, the final epitope boundary was defined by the exact contiguous segment shared by the consensus predictions; for instance, if one tool predicted ABCDEFG and another predicted CDEFGH, the resulting consensus epitope was defined as the common segment CDEFG. Finally, to ensure potential immunological relevance and exclude trivially short overlaps, a minimum length filter of five amino acids was applied, and only sequences meeting this criterion were retained as final candidate epitopes [32,37].

### 2.5. Prediction of the Tertiary Structure of Sesame Allergenic Proteins

The three-dimensional structures of the sesame allergenic protein targets were predicted using the AlphaFold 3 (AF3) model, which is the only unified deep-learning model for highly accurate biomolecular complexes from proteins to nucleic acids, small molecules, ions and modified residues [27]. The target of each protein was defined by only its protein amino acid sequence. AF3 uses a more concise module and a diffusion model to directly predict atomic coordinates for any chemical composition, moving beyond the limitations of AF2. The model was run with five independent random seeds, and each generated five diffusion samples for a total of 25 alternative structures per target. We ranked predicted structures by internal confidence values from the model. For the global ranking, we combined the predicted TM score (pTM) and interface predicted TM score (ipTM) with penalty terms that decrease the number of clashes when those numbers become extremely large and with penalty terms that incentivize disorder as shown to promote better folding [38]. Overall Confidence Score The high-scoring, top-ranked prediction was used as the final predicted tertiary structure for all analyses, with local accuracy determined through use of the predicted Local Distance Difference Test (pLDDT) [27].

## 3. Results and Analysis

### 3.1. Prediction of Linear B-Cell Epitopes in Sesame Allergenic Proteins

#### 3.1.1. Basic Physicochemical Properties of Sesame Allergenic Proteins

The average GRAVY values for the seven sesame allergenic proteins were found to be −0.808, −0.589, −0.902, and −0.537 for Ses i 1, Ses i 2, Ses i 6, and Ses i 7, indicating their hydrophilic nature. In contrast, Ses i 4 and Ses i 5 had average GRAVY values of 0.199 and 0.296, suggesting their hydrophobic character.

#### 3.1.2. Prediction of Linear B-Cell Epitopes Using DNAStar

**Figure 1:**
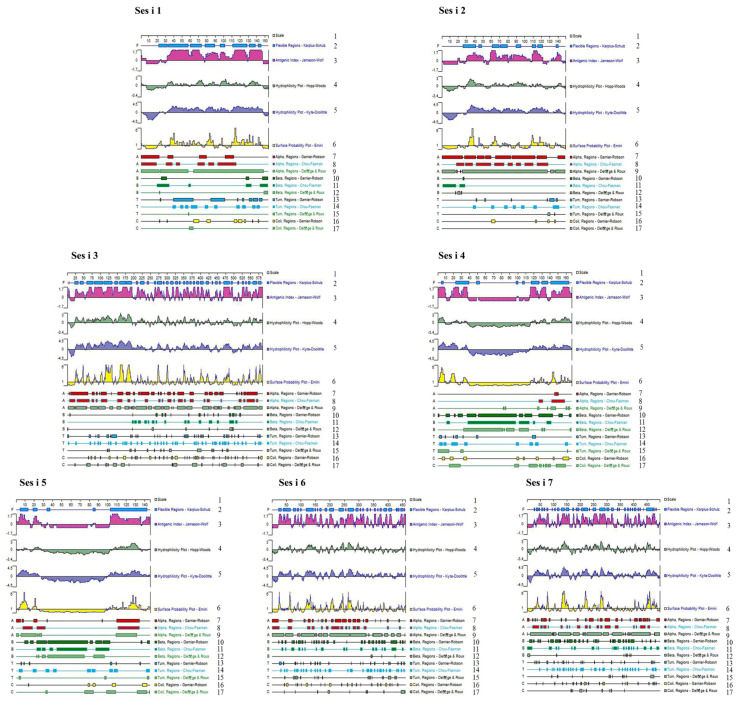
Prediction of flexibility, antigenicity, hydrophilicity, surface probability and secondary structure of sesame allergen Ses i 1−7 using DNAStar.

Linear epitopes, which are essential components of antigens, are typically exposed on the surface of allergenic molecules. They are located within flexible regions and consist of polar amino acid residues. These epitopes typically comprise 5 to 7 amino acids but do not exceed 30 amino acids. In our analysis, DNAStar software suite v7.1 (DNASTAR Inc., Madison, WI, USA) was employed to predict the antigenic index, hydrophilicity, surface accessibility, and flexibility for Ses i 1 to Ses i 7. These prediction results are visually depicted in Figure 1. Hydrophilicity indicates the ability of a protein to attract water molecules or dissolve in water. Flexibility refers to the ability of a protein to stretch physical traits by physical means. Surface accessibility is the likelihood of amino acid residues in a protein coming into contact with solvent molecules. The antigenicity index is the region of a protein where antigenic epitopes are likely to be present, as predicted by the DNAStar software using the Jameson–Wolf algorithm. Regions with a surface accessibility index > 1 and an antigenicity index > 0 were clustered, indicating that the area was highly susceptible to epitope formation [39,40,41,42]. Our B-cell epitopes selection criteria comprise regions exhibiting positive hydrophilicity, positive antigenic indices, surface accessibility values greater than 1, and flexibility, as indicated by the overlaid rectangles. These areas possess favorable hydrophilic properties, ease of folding and unfolding, and are prone to exposure, thereby enhancing their epitope-forming capacity.

From the graphical representations, it is evident that regions with a high flexibility index, as indicated by the overlaid rectangles, correspond to areas that exhibit a dominant potential for linear epitopes. With the exception of Ses i 4 and Ses i 5, the remaining five allergenic proteins display relatively uniform distributions of high flexibility and antigenic indices. Ses i 3, Ses i 6, and Ses i 7 demonstrate well-distributed regions with good hydrophilicity. Furthermore, the two ends of Ses i 4 and Ses i 5 show areas with good surface accessibility. Among other proteins, surface accessibility is relatively evenly distributed.

Additionally, the secondary structure of proteins plays a significant role in epitope prediction. α-helical and β-sheet structures are less likely to deform and are less conducive to antibody binding. In contrast, β-turns and random coils are structurally protruding regions that readily interact with antibodies, thereby increasing the likelihood of amino acid sequences in these areas serving as potential antigenic epitopes. Our secondary structure predictions for protein epitopes were conducted using DNAStar subprograms, employing the Chou-Fasman, Gamier-Robson, and Deleage-Roux algorithms. The results for secondary structure prediction for the seven proteins are also presented visually in Figure 1. In this figure, regions identified as β-turns (T) or random coils (C) are uniformly distributed across the entire amino acid sequences, indicating a heightened probability of linear epitope occurrence in these regions. Consequently, taking into consideration the hydrophilicity index, antigenic index, surface accessibility index, flexibility index, and secondary structure predictions by DNAStar, we present the anticipated linear B-cell epitopes in Table 1.

#### 3.1.3. Prediction of B-Cell Linear Epitopes of Sesame Allergenic Proteins Using SOPMA

Utilizing the SOPMA web server for secondary structure prediction of these proteins, the statistics regarding the composition of amino acids forming α-helices, β-sheets, β-turns, and random coils were analyzed, as shown in Table S1. The results indicate that over 50% of amino acid residues in Ses i 6 and Ses i 7 form β-turns and random coils, making these regions predominant for antigenic epitopes. Following these, Ses i 3, Ses i 4, and Ses i 5 exhibit approximately 40% of β-turns and random coils. Based on this, linear epitopes within sesame allergenic proteins were predicted, as shown in Table 2.

**Table 2:** Prediction of B-cell epitopes using SOPMA.

Sesame Allergen	Source (Position)
Ses i 1	21~24, 33~34, 38~39, 50~52, 61~70, 78~85, 117~122, 134~149
Ses i 2	22~24, 31, 62~67, 75~79, 94~97, 133~144
Ses i 3	2~5, 24~29, 61~72, 82~85, 94~96, 99~102, 119~133, 142~151, 167~175, 179~181, 189~195, 199~201, 208~213, 240~244, 248~254, 261~264, 270~275, 280~283, 288~291, 297~302, 311~319, 325~330, 348~349, 359~363, 368~369, 381~394, 399~410, 414~420, 434~438, 441~449, 456~460, 465~483, 492~496, 501~505, 511~515, 524~530, 535~536, 553~555, 566~569, 574~584
Ses i 4	2~37, 47~51, 57~58, 71~73, 78~84, 95, 98~103, 120~123, 157~160
Ses i 5	5~15, 18~21, 33~37, 43~45, 47~49, 56~58, 63~70, 79~80, 85~88, 103~110, 135~145
Ses i 6	22~32, 41~54, 59~62, 77~92, 98~101, 107~140, 147~151, 156~159, 165~170, 178~183, 187~191, 197~213, 220~221, 231~232, 242~247, 253~256, 260~264, 268~275, 291~298, 302~307, 312~317, 332~335, 338~346, 353~356, 363~366, 373~377, 382~384, 391~396, 403~407, 414~415, 421~423, 433~434, 444~448, 453~459
Ses i 7	27~29, 36~47, 56~69, 74~78, 93~107, 113~117, 122~142, 145~146, 154~157, 162~165, 171~176, 184~190, 195~196, 203~225, 231~233, 243~244, 253~259, 265~268, 271~290, 303~310, 314~319, 324~329, 339~340, 344~347, 350~358, 365~368, 375~378, 385~389, 394~396, 403~409, 414~419, 427~428, 434~436, 446~447, 457~460, 466~485

#### 3.1.4. Prediction of Sesame Allergenic Protein B-Cell Linear Epitopes Using BepiPred 1.0

The allergenic protein sequences were submitted to the BepiPred web server, which uses a Markov model and a scale method to predict B-cell linear epitopes. BepiPred is a versatile tool encompassing various functionalities, such as biological sequence alignment, homology analysis, gene identification, protein secondary structure prediction, signal peptide prediction, and antigenic epitope prediction. The results are presented in Table 3.

**Table 3:** Prediction of B-cell epitopes using BepiPred.

Sesame Allergen	Source (Position)
Ses i 1	22~45, 61~75, 78~87, 112~130, 134, 140~142
Ses i 2	29~46, 63~65, 74~79, 112~118, 120, 138, 140
Ses i 3	22~31, 38, 40~49, 62~75, 80~86, 94~104, 113~134, 143~152, 155~193, 240, 243~245, 250~253, 274, 312~318, 325~332, 370~373, 378~394, 400~409, 411, 413~414, 416~417, 436~447, 460, 468~487, 511, 513, 525~533, 545, 556~567, 575~585
Ses i 4	1~25, 29~38, 119~135, 138~166
Ses i 5	3~13, 23, 31~34, 104~116, 118~122, 125~145
Ses i 6	21~31, 40~60, 62~64, 77~91, 111~113, 116, 121~137, 154~155, 157, 164~170, 198~211, 230~232, 234, 236~245, 259~278, 293~296, 326~327, 329~331, 336~338, 340~343, 354~358, 363, 373~374, 384~395, 403~413, 431~440, 455~459
Ses i 7	24~27, 29~30, 35~43, 55~64, 67~68, 74~75, 77~80, 103~104, 116~118, 122~124, 127~139, 172~176, 186~191, 204~227, 250~257, 272~291, 303~320, 378~379, 402~408, 416~422, 446~451, 4576~459, 461~464, 467~484

**Figure 2:**
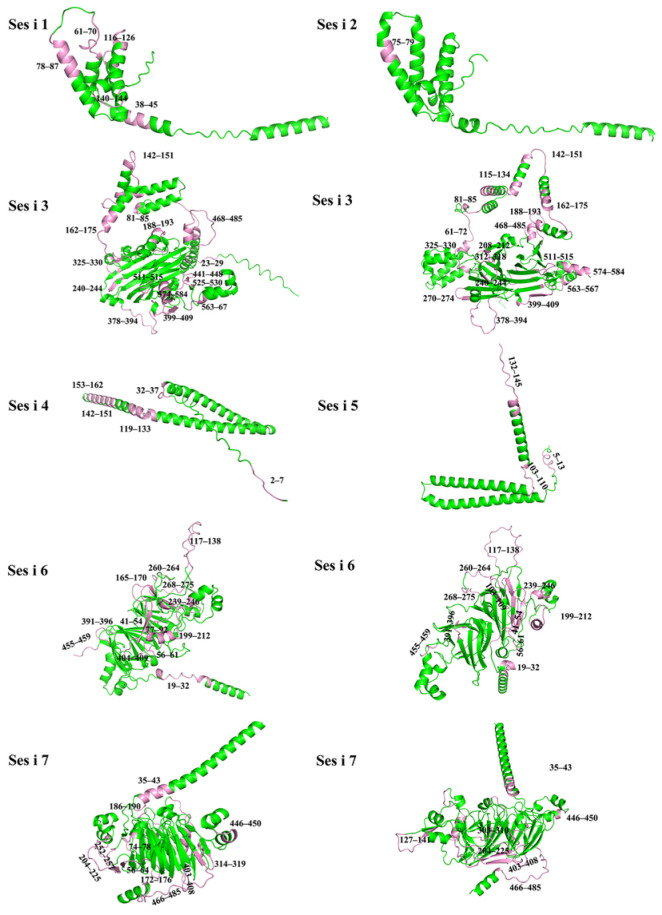
Spatial conformation of predicted B cell linear antigenic epitopes in sesame allergens. The predicted epitopes are highlighted in pink, and the numbers indicate their corresponding positions in the amino acid sequence.

Comparing the results obtained from these three prediction methods reveals a general consistency in their predictions. Combining the results using the approach outlined in reference, we selected regions predicted by two or more methods as the final linear epitope regions [24]. This approach not only enhances the accuracy of the predictions but also ensures a broader selection of prominent linear epitope regions. The details of the predicted epitope sequences are presented in Table 4, as well as experimental epitopes. The spatial distribution of some of the predicted B-cell linear epitopes is depicted in Figure 2 and beyond, providing multiple perspectives. We found that random coils are the predominant structure, accounting for an average of 74.1% of the epitope regions, presented in Table S2.

**Figure 3:**
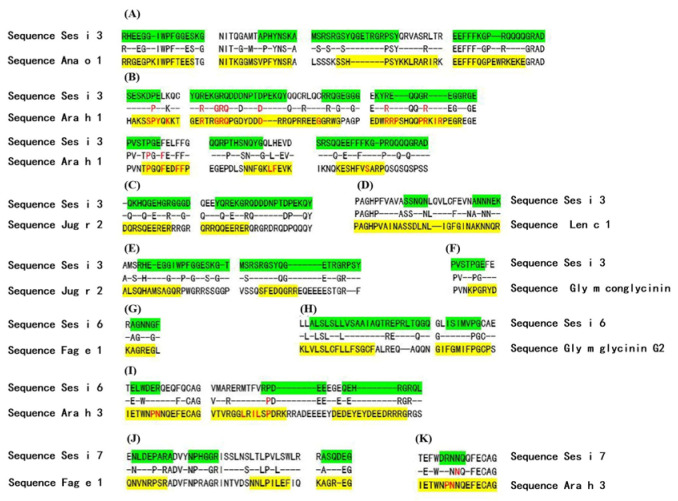
Sequence alignment of sesame allergens from the Cupin superfamily. Predicted B-cell epitopes are highlighted in green. Experimentally known IgE-binding epitopes are highlighted in yellow (retrieved from the IEDB), with their critical binding residues shown in red font. (**A**): Ses i 3 and Ana o 1; (**B**): Ses i 3 and Ara h 1; (**C**): Ses i 3 and Jug r 2; (**D**): Ses i 3 and Len c 1; (**E**): Ses i 3 and Jug r 2; (**F**): Ses i 3 and Gly m conglycinin; (**G**): Ses i 6 and Fag e 1; (**H**): Ses i 6 and Gly m conglycinin G2; (**I**): Ses i 6 and Ara h 3; (**J**): Ses i 7 and Fag e 1; (**K**): Ses i 7 and Ara h 3.

Multiple sequence alignment revealed a high degree of homology between the subject protein and other allergens within the same family (Figure 3). More importantly, this conservation extends to the epitope level. Our analysis identified that 28 of the 62 predicted epitopes are located in regions that are positionally conserved with previously reported epitopes in homologous allergens [43,44,45,46,47]. The conservation of key amino acid residues within these regions not only corroborates the predictive accuracy of our bioinformatics approach but also suggests a potential for immunological cross-reactivity. Notably, no experimental antigenic epitopes have been previously reported for Ses i 1, 4, 6, and 7, making our predictions for these allergens entirely novel. For Ses i 3, five of our predicted epitopes overlapped with known experimental data, while the remaining 15 represent new potential epitopes. Detailed information on this comparison is provided in Supplementary Table S3.

Furthermore, through comparative analysis of known allergenic binding epitopes in homologous allergenic proteins within the same family, the predicted epitope RHEEGGIWPFGGESKGT of Ses i 3 shares up to 75% identity (Table S5) with the peptide segment RRGEGPKIWPFTEES found in Ana o 1 (a known IgE-binding allergenic epitope). Cross-reactivity occurs between the IgE antibodies of both allergens [45]. In addition, Ses i 6 and Ses i 7 share 39% overall homology with Ara h 3 (an 11S globulin found in peanuts), with significant homology in corresponding regions to known IgE-binding allergenic epitopes in Ara h 3 [37,38]. There are also numerous similarities in key IgE recognition sites between the two (Figure 3, Table S3).

**Table 4:** Predicted allergenic epitopes from this study and their comparative analysis with known experimentally validated epitopes.

Sesame Allergen	Peptide	Source (Position)	Number of Peptides	Experimental Epitopes Position
Ses i 1	NQQSQQCR, SQGRSPYGGE, STGNQQSEQS, QEGGYQEGQSQ, NMRPQ	38~45, 61~70, 78~87, 116~126, 140~144	5	
Ses i 2	ANQGQ	**75~79**	1	46–55, 48–57, **76–86** [19]
Ses i 3	SESKDPE, QKHQGEHGRGGG, NRKSP, YQREKGRQDDDNPTDPEKQY, RRQGEGGGFS, KYREQQGREGGRGE, EQGRGR, RTQHG, AEPQT, RQDRR, PVSTPGE, AGGENP, RHEEGGIWPFGGESKGT, QQRPTHSNQYG, APHYNSKA, MSRSRGSYQGETRGRPSY, SSNQN, ANNNEK, SRSQQ, GPRQQQQGRAD	23~29, 61~72, 81~85, 115~134, 142~151, 162~175, 188~193, 208~212, 240~244, 270~274, 312~318, 325~330, 378~394, 399~409, 441~448, 468~485, 511~515, 525~530, 563~567, 574~584	20	
Ses i 4	ADRDRP, **QKGPST**, **RATGQGPLEYAKRGV**, EKTKQAGEAI, STAKEGGREG	2~7, **32~37, 119~133**, 142~151, 153~162	5	**35–40,** 98–106, **130–139** [20]
Ses i 5	**YGQQQQTRA, TGKHPPGA, EQFSQQPVAGSQTS**	**5~13, 103~110, 132~145**	3	**1–15,** 11–25, 61–75, 71–85, **101–115,** 111–125, **131–145** [45]
Ses i 6	AIAQTREPRLTQGQ, GAQPSLRIQSEGGT, ELWDER, IRPNGLSLPNYHPSPR, ISIMVPG, HRSQRTMERTEASEQQDRGSVR, NDGSED, VPRSGEQEQQARQT, MQSEEEER, RPDEE, QEHRGRQL, AGNNGF, TGSPMR, GGRRS	19~32, 41~54, 56~61, 77~92, 103~109, 117~138, 165~170, 199~212, 239~246, 260~264, 268~275, 391~396, 404~409, 455~459	14	
Ses i 7	LQSQQQHKL, AQEPTIRFE, DRNNQ, ETFERDTQPRQDRRR, NGGEP, GNAAN, NPQGGRQSYFGRPQTEKQQGET, KGQDDL, PGEEEEERWERDPYSGANG, NLDEPARA, NPHGGR, ASQDEG, VSRDE, STSRYSWPRSSRPMSYMPKP	35~43, 56~64, 74~78, 127~141, 172~176, 186~190, 204~225, 252~257, 272~290, 303~310, 314~319, 403~408, 446~450, 466~485	14	

Boldface denotes overlap with known experimental epitopes.

### 3.2. Homology Modeling and Quality Assessment of Sesame Allergens

In order to give a structural basis for the sesame allergenicity, the tertiary structures of the seven main sesame allergens (Ses i 1 to Ses i 7) have been predicted with the AF3 server. The quality of the models generated was evaluated with the average per-residue confidence score (pLDDT; we refer to pLDDT as the ranking score) and the predicted Template-Modeling (pTM) score, which examines the quality of the global domain packing. As summarized in Table 5, most of the allergens produced high-confidence models. Among these, the structures for Ses i 6 and Ses i 7 were predicted with high reliability (0.92 and 0.87 pLDDT values and 0.84 and 0.82 pTM values, respectively). Good quality models were predicted for Ses i 1 (pLDDT = 0.79, pTM = 0.63), Ses i 3 (pLDDT = 0.78, pTM = 0.66), and Ses i 2 (pLDDT = 0.74, pTM = 0.62). It was observed that the six structures (model i61) were of good quality, also indicating that it is an approximately good model that could be used for further analysis. For the two models, Ses i 4 and Ses i 5, however, the confidence was much worse with pLDDT of 0.58 and 0.67 and specifically the low pTM scores of 0.30 and 0.31, respectively. These lower scores imply that these two proteins either have significant disordered areas or that their predicted inter-domain orientation is of low fidelity and should be treated with caution in their interpretation (see Table 5).

**Figure 4:**
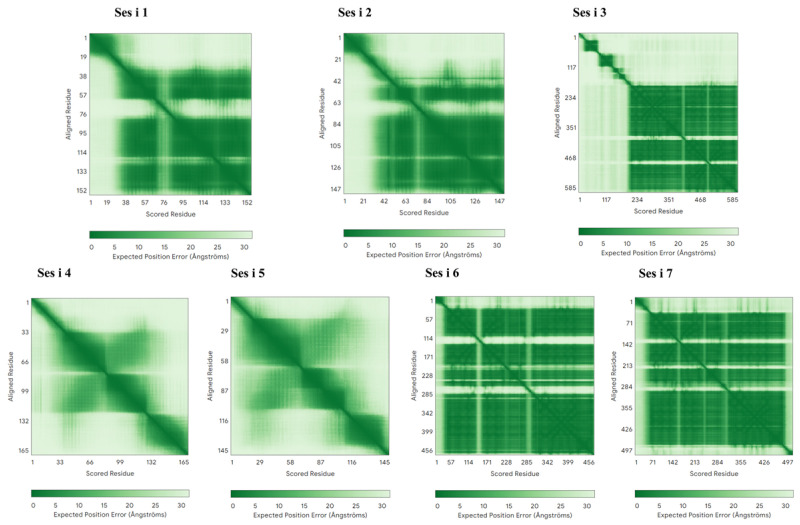
AlphaFold3 predicted aligned error (PAE) for the structure of sesame allergens (darker is more confident).

The corresponding predicted aligned error (PAE) plots are presented in Figure 4. The PAE matrices provide a quantitative assessment of the confidence in the relative positioning of residues within each protein model, with lower PAE values (darker regions) indicating higher structural reliability. For Ses i 1, Ses i 2, Ses i 3, Ses i 6, and Ses i 7, the PAE plots display predominantly low error values along the diagonal, suggesting well-defined tertiary structures and reliable domain organization. In contrast, Ses i 4 and Ses i 5 exhibit regions of increased PAE, particularly in off-diagonal areas, which may reflect flexible segments, inter-domain variability, or regions of lower model confidence. Overall, the generation of high-confidence structural models for the majority of key sesame allergens via AlphaFold3 not only establishes a robust foundation for mapping and visualizing predicted epitopes but also highlights regions of structural uncertainty, thereby providing critical guidance for future experimental validation.

## 4. Discussion

The accurate identification of B-cell linear epitopes is paramount for advancing our understanding of allergenicity and for the rational design of targeted immunotherapeutic and diagnostic strategies. Our study employed a comprehensive multi-tool bioinformatics approach, integrating DNAStar, SOPMA, and BepiPred-1.0, to predict B-cell linear epitopes across seven major allergenic sesame proteins. This methodology is predicated on the established principles that epitope formation is significantly influenced by a combination of physicochemical properties, including secondary structure, hydrophilicity, surface accessibility, antigenicity, and flexibility. Hydrophilic regions and random coil regions are more likely to be epitopes due to their high surface accessibility and flexibility [46]. Robust immunological responses are expected from the epitopes displaying strong potential of binding. Conversely, stable higher-order structures like α-helices and β-sheets, often buried within the protein core, present reduced accessibility for antibody binding. Our finding that the majority of epitopes reside in random coils is highly significant and consistent with established principles of immunogenicity. By systematically integrating these parameters, our approach aimed to identify regions with the highest propensity for B-cell recognition.

Our analysis predicted a total of 62 potential B-cell linear epitopes among the seven sesame allergens, with Ses i 3 exhibiting the highest number (20 epitopes). We identified that 28 of the 62 predicted epitopes are located in regions that are positionally conserved with previously reported epitopes in homologous allergens, sharing certain key amino acids. This initial concordance provides a degree of in silico validation for our prediction methodology, suggesting its utility in identifying immunologically relevant regions.

To further substantiate the validity of our predictions and contextualize them within the existing body of knowledge, we conducted a detailed comparative analysis with the only two published experimental studies characterizing B-cell epitopes in Ses i 2 and Ses i 4 [19,20]. For Ses i 2, our predicted linear epitope (residues 75–79) showed sequence overlap with the experimentally validated epitopes reported by Marchand, C. at positions 76–86 [19]. This strong agreement highlights the predictive power of our integrated bioinformatics approach. Similarly, for Ses i 4, QKGPST, and RATGQGPLEYAKRGV, two of our predicted epitopes (residues 32–37 and 119–133) corresponded well with the experimentally determined regions by Wolff, N at (35–40 and 130–139). Our predicted epitope showed 50% overlap with the experimentally identified epitope. While some discrepancies were observed, possibly attributable to differences in experimental methodologies or the inherent limitations of linear epitope prediction versus conformational epitopes, the overall consistency provides robust support for the biological relevance of our in silico findings. This direct comparison not only validates our predictions against empirical data but also helps to prioritize the most promising candidates for future experimental investigation.

Furthermore, our team has recently identified seven B-cell epitopes for Ses i 5 through experimental validation using overlapping peptide arrays. This direct experimental evidence provides a crucial benchmark, allowing us to directly assess the accuracy and relevance of our bioinformatics approach for this specific allergen [47]. Our in silico predictions for Ses i 5 demonstrated a substantial overlap with these experimentally determined epitopes. Specifically, three of our predicted epitopes (located at residues 5–13, 103–110, and 132–145) showed high concordance with the experimentally validated regions (1–15, 101–115, and 131–145, respectively). This strong agreement between computational prediction and experimental validation significantly strengthens the confidence in our predictive models across the entire panel of sesame allergens.

Beyond linear epitope prediction, our study also incorporated advanced structural modeling. Initially, homology modeling was performed using SWISS-MODEL. However, to leverage the latest advancements in protein structure prediction and enhance the accuracy of our models, we subsequently employed AlphaFold3 for all seven sesame allergens. This allowed us to generate highly reliable tertiary structures, which are critical for providing a spatial context to the predicted linear epitopes. By visualizing these epitopes on the protein’s 3D surface, we can assess their surface accessibility and conformational flexibility, both of which are paramount for effective antibody binding. For instance, QKHQGEHGRGGG, RRQGEGGGFS, KYREQQGREGGRGE, EQGRGR, RHEEGGIWPFGGESKGT, and MSRSRGSYQGETRGRPSY (61~72, 142~151, 162~175, 188~193, 378~394, 468~485), our AlphaFold3 models revealed that several highly antigenic linear epitopes predicted for Ses i 3 are indeed located on exposed loop regions, suggesting their high likelihood of being recognized by B-cells in their native folded state. Conversely, predicted epitopes located within deeply buried α-helical or β-sheet structures, despite high antigenicity scores, are less likely to be immunologically relevant due to steric hindrance. The structural information aids in the selection of spatially accessible epitopes, thereby improving our understanding of allergen recognition and detection beyond a purely sequence-based perspective.

Although the oleosin proteins (Ses i 4 and Ses i 5) are largely buried within oil bodies, our analysis included the entire protein sequence. This is because food processing (e.g., roasting, grinding) and digestion in the gastrointestinal tract are known to cause significant protein denaturation and structural disruption. These events can expose previously inaccessible polypeptide regions to the immune system, making them immunologically relevant [48].

While our in silico approach offers significant advantages in terms of speed and cost-effectiveness for initial epitope identification and prioritization, it is important to acknowledge its inherent limitations. Bioinformatic tools predict potential epitopes based on algorithms and physicochemical properties, but they cannot fully replicate the complex biological environment of antibody–antigen interactions, which often involve conformational epitopes and dynamic binding events. Therefore, the ultimate confirmation of the allergenicity and immunogenicity of these predicted epitopes necessitates rigorous experimental validation through further in vitro and in vivo studies.

It is important to note that the current study is primarily based on in silico predictions. While these computational analyses offer invaluable insights and significantly narrow down the candidates for further investigation, the ultimate confirmation of epitope allergenicity and immunogenicity necessitates rigorous experimental validation. Our detailed structural and epitope predictions serve as a critical guide for designing such future in vitro and in vivo experiments.

## 5. Conclusions

The present study addresses a critical gap in understanding sesame allergens, which have garnered significant attention due to their increasing prevalence and potential to cause severe allergic reactions, including anaphylaxis. Despite the identification of seven major sesame allergens, detailed investigations into their structures and epitopes have been limited. By utilizing robust immunoinformatic tools, we successfully predicted the tertiary structures of these allergens and 62 potential B-cell epitopes, with 28 peptides demonstrating partial sequence overlap with known epitopes from related allergenic proteins. They represent high-value candidates for future experimental validation. The spatial distribution of these epitopes was further elucidated, providing a comprehensive view of their potential immunogenicity. These findings not only enhance our understanding of sesame allergenicity but also offer valuable insights for the development of immunotherapeutic vaccines. The predicted epitopes and structures identified in this study are proposed as suitable candidates for the design of targeted immunotherapies, potentially revolutionizing the management of sesame allergies. Future research should focus on the experimental validation of these predictions to confirm their efficacy and applicability in clinical settings.

## Figures and Tables

**Table 1:** Prediction of B-cell epitopes using DNAStar.

Sesame Allergen	Source (Position)
Ses i 1	39~49, 5~52, 56, 60~70, 73, 78~81, 83, 85~87, 96~98, 100, 116~119, 123~126, 131~135, 138, 142~144
Ses i 2	38~40, 61~64, 76, 78~79, 111~113, 139
Ses i 3	23, 25~28, 60~68, 81~85, 92, 96, 99~101, 115~135, 142~145, 151, 162~172, 174~175, 178~180, 186, 188~191, 193, 207~212, 220~225, 240~242, 262, 270~273, 280, 298~300, 316~317, 328~330, 332, 348~353, 359~361, 378~380, 390~392, 399~408, 416~418, 423, 445~448, 469~485, 492~493, 512~515, 525~529, 535~537, 563~566, 573~582
Ses i 4	5~7, 29~30, 32~37, 117~121, 125, 132, 151, 153~162
Ses i 5	5~7, 9, 19~22, 103~109, 132~135, 142
Ses i 6	25~30, 32, 42, 44, 51~53, 56, 75~78, 87~90, 99, 118~123, 133~136, 148, 166~168, 185, 201~203, 239~241, 261~263, 268, 270~275, 278, 290, 292, 305, 312~315, 327~330, 352, 354, 364, 372, 374, 385, 391~392, 404, 406~409, 429, 433~434, 436~437, 444~447, 455~456
Ses i 7	29, 34~38, 40, 45~47, 60, 74~78, 104~105, 115, 131~141, 146~149, 154, 172~173, 206~212, 214~219, 221~223, 232, 252~257, 273~274, 280~286, 307~308, 315~316, 366, 376, 404~407, 415~419, 447~450, 456~457, 461, 466~470, 473~478, 483~485

**Table 5:** Summary of AlphaFold3 prediction parameters and quality metrics for sesame allergen structures.

Sesame Allergens	Ranking Score (pLDDT)	pTM
Ses i 1	0.79	0.63
Ses i 2	0.74	0.61
Ses i 3	0.78	0.66
Ses i 4	0.58	0.3
Ses i 5	0.67	0.31
Ses i 6	0.92	0.84
Ses i 7	0.87	0.82

## Data Availability

The original contributions presented in this study are included in the article/Supplementary Materials. Further inquiries can be directed to the corresponding author.

## Supplementary Materials

The following supporting information can be downloaded at: https://www.mdpi.com/article/10.3390/foods14234158/s1, Table S1. Secondary structure prediction of of sesame allergens using SOPMA. Table S2. Secondary structure proportion of predicted epitopes. Table S3. Experimentally known B-cell epitopes are highlighted in green (retrieved from the IEDB), predicted B-cell epitopes are shown in red font. Table S4. Experimental epitopes of other, homologous allergens Immune Epitope Database. Table S5. Scoring Table for Sequence Alignment.
