# Supplementary material for: Homology Modeling of Sesame Allergenic Protein and Prediction of B-Cell Linear Antigenic Epitopes Using Immunoinformatic Tools
Source: Foods. 2025 Dec 3;14(23):4158. doi: 10.3390/foods14234158 (PMC12692619; doi:10.3390/foods14234158)
Supplement: Supplementary file 1 [file foods-14-04158-s001.zip › foods-3950157-supplementary.pdf]

Supplemental Files

Table S1. Secondary structure prediction of of sesame allergens using SOPMA.

Allergens	α-helix		β-sheet		β-turn		Random coil	
	Proportion (%)	Number of amino acids	Proportion (%)	Numberof amino acids	Proportion (%)	Numberof amino acids	Proportion (%)	Number of amino acids
Ses i 1	56.21	86	10.46	16	3.27	5	30.07	46
Ses i 2	72.97	108	6.08	9	4.05	6	16.89	25
Ses i 3	36.75	215	18.97	111	9.91	58	34.36	201
Ses i 4	43.37	72	15.66	26	5.42	9	35.54	59
Ses i 5	38.62	56	18.62	27	11.72	17	31.03	45
Ses i 6	29.63	136	19.83	91	7.63	35	42.92	197
Ses i 7	29.58	147	19.72	98	7.04	35	43.66	217

Table S2. Secondary structure proportion of predicted epitopes.

Allergens	α-helix		β-sheet		β-turn		Random coil		Extended strand		Total AA count of epitopes
	Proportion (%)	AA count	Proportion (%)	AA count	Proportion (%)	AA count	Proportion (%)	AA count	Proportion (%)	AA count	
Ses i 1	29.5	13	0	0	9.1	4	61.4	27	0.0	0	44
Ses i 2	0.0	0	0	0	0.0	0	100.0	5	0.0	0	5
Ses i 3	11.5	21	0	0	19.1	35	69.4	127	0.0	0	183
Ses i 4	57.4	27	0	0	4.3	2	38.3	18	0.0	0	47
Ses i 5	9.7	3	0	0	16.1	5	74.2	23	0.0	0	31
Ses i 6	6.9	9	0	0	9.2	12	84.0	110	4.6	6	131
Ses i 7	3.6	5	0	0	5.0	7	91.4	128	0.0	0	140
Average	16.9	11	0.0	0	9.0	9	74.1	63	0.7	1	83

Table S3. Experimentally known B-cell epitopes are highlighted in green (retrieved from the IEDB), predicted B-cell epitopes are shown in red font.

**Sesame allergen**	**amino acid sequence (position)**
Ses i 1 (Q9AUD1)	MAKKLALAAVLLVAMVALASATTYTTTVTTTAIDDEANQQSQQCRQQLQGRQFRSCQRYLSQGRSPYGGEEDEVLEMSTGNQQSEQSLRDCC<br>QQLRNVDERCRCEAIRQAVRQQQQEGGYQEGQSQQVYQRARDLPRRCNMRPQQCQFRVIFV
Ses i 2 (Q9XHP1)	MARFTIVLAVLFAAALVSASAHKTVVTTSVAEEGEEENQRGCEWESRQCQMRHCMQWMRSMRGQYEESFLRSAEANQGQFEHFRECCN<br>ELRDVKSHCRCEALRCMMRQMQQEYGMEQEMQQMQQMMQYLPRMCGMSYPTECRMRPIFA
Ses i 3 (Q9AUD0)	MSCGGRLCLVLFALLLASAVVASESKDPELKQCKHQCKAQQQISKEQKEACIQACKEYIRQKHQGEHGRGGGDILEEEVWNRKSPIERLR<br>ECSRGCEQQHGEQREECLRRCQEEYQREKGRQDDDNPTDPEKQYQQCRLQCRRQGEGGGFSREHCERRREEKYREQQGREGGRGEMYEGRE<br>REEEQEEQGRGRIPYVFEDQHFITGFRTQHGRMRVLQKFTDRSELLRGIENYRVAILEAEPQTFIVPNHWDAESVVFVAKGRGTISLVRQDRRESL<br>NIKQGDILKINAGTTAYLINRDNNERLVLAKLLQPVSTPGEFELFFGAGGENPESFFKSFSDEILEAAFNTRRDRLQRIFGQQRQGVIVKASEEQV<br>RAMSRHEEGGIWPFGGESKGTINIYQQRPTHSNQYGQLHEVDASQYRQLRDLDLTVSLANITQGAMTAPHYNSKATKIALVVDGEGYFEMAC<br>PHMSRSRGSYQGETRGRPSYQRVASRLTRGTVVIIPAGHPFVAVASSNQNLQVLCFEVNANNNEKFPLAGRRNVMNQLEREAKELAFGMPAR<br>EVEEVSRSQQEEFFFKGPRQQQQGRADA
Ses i 4 (Q9FUJ9)	MADRDRPHPHQIQVHPQHPHRYEGGVKSLLPQKGPSTTQILAIITLLPISGTLLCLAGITLVGTLIGLAVATPVFVIFSPVLVPAAILIAGAVTAFLT<br>SGAFGLTGLSSLSWVLNSFRRATGQGPLEYAKRGVQEGTLYVGEKTKQAGEAIKSTAKEGGREGTART
Ses i 5, (Q9XHP2)	MAEHYGQQQQTRAPHLQLQPRAQRVVKAATAVTAGGSLLVLSGLTLAGTVIALTIATPLLVIFSPVLVPAVITIFLLGAGFLASGGFGVAALSVLS<br>WIYRYLTGKHPPGADQLESAKTKLASKAREMKDRAEQFSQQPVAGSQTS
Ses i 6 (Q9XHP0)	MVAFKFLLALSLSLLVSAAIAQTREPRLTQGQQCRFQRISGAQPSLRIQSEGGTTELWDERQEQFQCAGIVAMRSTIRPNGLSLPNYHPSPRLVYI<br>ERGQGLISIMVPGCAETYQVHRSQRTMERTEASEQQDRGSVRDLHQKVHRLRQGDIVAIPSGAAHWCYNDGSEDLVAVSINDVNHLSNQLD<br>QKFRAFYLAGGVPRSGEQEQQARQTFHNIFRAFDAELLSEAFNVPQETIRRMQSEEEERGLIVMARERMTFVRPDEEEGEQEHRGRQLDNGLE<br>ETFCTMKFRTNVESRREADIFSRQAGRVHVVDRNKLPILKYMDLSAEKGNLYSNALVSPDWSMTGHTIVYVTRGDAQVQVVDHNGQALMN<br>DRVNQGEMFVVPQYYTSTARAGNNGFEWVAFKTTGSPMRSPLAGYTSVIRAMPLQVITNSYQISPNQAQALKMNRGSQSFLLSPGGRRS
Ses i 7 (Q9AUD2)	MALTSLLSFFIVVTLLIRGLSAQLAGEQDFYWQDLQSQQQHKLQARTDCRVERLTAQEPTIRFESEAGLTEFWDRNNQQFECAGVAAVRNVIQ<br>PRGLLLPHYNNAPQLLYVVRGRGIQGTVIPGCAETFERDTQPRQDRRRRFMDRHQKVRQFRQGDILALPAGLTLWFYNNGGEPLITVALLDT<br>GNAANQLDQTFRHFFLAGNPQGGRQSYFGRPQTEKQQGETKNIFNGFDDEILADAFGVDVQTARRLKGQDDLRGRIVRAERLDIVLPGEEEE<br>ERWERDPYSGANGLEETLCTAKLRENLDEPARADVYNPHGGRISSLNSLTLPVLSWLRLSAEKGVLYRNGLVAPHWNLNAHSIIYITRGSGRF

QVVGHTGRSVFDGVVREGQLIIVPQNYVVAKRASQDEGLEWISFKTNDNAMTSQLAGRLSAIRAMPEEVVMTAYQVSRDEARRLKYNREESR
VFSSTSRYSWPRSSRPMSYMPKPFEYVLDVIKSMM

Table S4. Experimental epitopes of other, homologous allergens Immune Epitope Database.

Allergen	Peptides
Ana o 1	RRGEGPKIWPFTEES, NITKGGMSVPFYNSR, SSHPSYKKLRARIR, EEFFFQGPEWRKEKE
Ara h 1	AKSSPYQKKT, GERTRGRQPGDYDDD, RRQPRREEGGRWG, EDWRRPSHQQPRKIRPEGR, TPGQFEDFFP, NNFGKLFEVK, KESHFVSARP
Jug r 2	DQRSQEERER, QRRQQEERER, ALSQHAMSAGQR, SFEDQGRR
Len c 1	PAGHPVAINASSDLNLIGFGINAKNNQR
Glym conglycinin	KPGRYD
Fag e 1	KAGREGL, QNVNRPSR, NNLPILEF, KAGREG
Gly m glycinin G2	KLVLSLCFLLFSGCF, GIFGMIFPGCPS
Ara h 3	IETWNPNNQEFECAG, VTVRGGLRILSPDRK, DEDEYEYDEEDRRRG

Table S5. Scoring Table for Sequence Alignment.

Highest Bit Score:	Total Bit Score:	Percent Coverage:	Ev alue:	Percent Identity:
21	21	47%	2e-05	75.00%

Sequence:RHEEGGIWPFGGESKGT(Ses i 3) and RRGEGPKIWPFTEES(Ana o 1)
